# The Army and Territorial Nurses' Cape

**Published:** 1915-10-30

**Authors:** 


### The Editor's Letter-Box.

### The Army and Territorial Nurses' Cape.

*To the Editor of* THE HOSPITAL.

SIR,—In your last week's number, so full of interesting articles on military hospital topics, I see one statement with which I fancy general agreement is not to be expected. I refer to the concluding paragraph on p. 74, wherein is extolled the Army nurses' cape. I have met a good many Army nurses "on duty" of both Regular and Territorial Q.A.I.M.N.S., and I do not remember hearing one of them say a good word for the cape. These ladies are precluded by the discipline of the service to which they belong from making public their opinion of the cape, which is part of their prescribed uniform. I believe if they were free to announce their views on the matter that the majority of them would advocate the abolition of this garment on grounds of its inconvenience and uselessness; perhaps, when the war is over, and many of them return to private life, they will tell you whether I am correctly representing them or not.—I remain, Sir, yours faithfully, CAPTAIN.

[The origin of the nurse's cape in question has been attributed to the late Florence Nightingale. As a matter of fact, it appears to have been settled between Miss Nightingale and her matrons, and was a matter which did not come before the Committee of the Nightingale Fund to decide, the Committee merely paying for the materials. Nothing is said about the cape in Sir Edward Cook's biography of Miss Nightingale, nor is it mentioned in the index.—ED. THE HOSPITAL.]

